# Epigenetic Mechanisms in Neurofibromatosis Types 1 and 2

**DOI:** 10.3390/epigenomes9030030

**Published:** 2025-08-14

**Authors:** Christina Stylianides, Gavriel Hadjigavriel, Paschalis Theotokis, Efstratios Vakirlis, Soultana Meditskou, Maria Eleni Manthou, Iasonas Dermitzakis

**Affiliations:** 1 Department of Histology-Embryology, School of Medicine, Aristotle University of Thessaloniki, 54124 Thessaloniki, Greece; christyl@auth.gr (C.S.); gavrchat@auth.gr (G.H.); ptheotokis@auth.gr (P.T.); sefthym@auth.gr (S.M.); mmanthou@auth.gr (M.E.M.); 2 First Department of Dermatology and Venereology, School of Medicine, Aristotle University of Thessaloniki, 54643 Thessaloniki, Greece; svakirlis@auth.gr

**Keywords:** neurofibromatosis type 1, neurofibromatosis type 2, phakomatoses, epigenetics, DNA methylation, histone modifications, non-coding RNAs

## Abstract

Neurocutaneous syndromes, known as phakomatoses, encompass a diverse group of congenital conditions affecting the nervous system and skin, with neurofibromatosis type 1 (NF1) and neurofibromatosis type 2 (NF2) among the most clinically significant. Both disorders are inherited in an autosomal dominant manner. NF1 presents with café-au-lait macules; cutaneous, subcutaneous, and plexiform neurofibromas; skeletal abnormalities; learning disabilities; and optic pathway gliomas, while NF2 is characterised by bilateral vestibular schwannomas, multiple meningiomas, ependymomas, and peripheral nerve schwannomas. Although germline mutations in the *NF1* and *NF2* tumour suppressor genes are well established, they do not fully explain the broad clinical variability observed, even among individuals carrying identical mutations. As increasingly recognised in other genetic diseases, epigenetic mechanisms, including DNA methylation, histone modifications, chromatin remodelling, and non-coding RNA (ncRNA) regulation, play a critical role in modulating gene expression and influencing disease severity. Despite important findings, the research remains fragmented, and a unified model is lacking. This review organises the current knowledge, emphasising how epigenetic alterations impact disease behaviour and outlining their potential as prognostic biomarkers and therapeutic targets. A deeper understanding of these mechanisms could lead to improved personalised management and the development of targeted epigenetic therapies for individuals with NF1 and NF2.

## 1. Introduction

Neurocutaneous syndromes represent a heterogeneous group of congenital disorders characterised by the simultaneous involvement of the nervous system and skin, originating from abnormalities in ectoderm-derived tissues [1,2]. They encompass a broad range of conditions, including neurofibromatosis type 1 (NF1), neurofibromatosis type 2 (NF2), tuberous sclerosis complex, Sturge–Weber syndrome, von Hippel–Lindau disease, and others [3]. Among these, neurofibromatoses hold particular clinical importance due to their multisystem involvement, potential for malignant transformation, and substantial impact on quality of life [4]. The heterogeneity of NF1 is reflected in its involvement of multiple organ systems beyond the nervous system and skin, including the heart, kidneys, lungs, and bones [5,6,7,8]. Similarly, NF2 exhibits significant clinical variability, with manifestations ranging from isolated vestibular schwannomas to extensive intracranial and spinal tumours leading to severe neurological deficits [9]. The high prevalence of NF1 further underscores its relevance in clinical practice and research, affecting approximately 1 in 2500 to 3500 individuals, whereas NF2 is considerably rarer, affecting approximately 1 in 50,000 individuals [10,11,12]. NF1 and NF2 have traditionally been considered monogenic disorders caused by mutations in the genes encoding neurofibromin and merlin, respectively [13,14]. Both exhibit complete penetrance, meaning that all individuals with a pathogenic variant eventually develop clinical features of the disease. However, the variable expressivity of these disorders results in a wide spectrum of manifestations, ranging from mild to severe, even among individuals carrying identical mutations [15,16,17]. This phenotypic heterogeneity, especially pronounced in NF1, suggests that additional mechanisms such as epigenetic modifications may contribute to disease development and expression [18,19].

Epigenetics refers to heritable changes in gene activity that occur without alterations to the DNA sequence, primarily through DNA methylation, histone modifications, and regulation by non-coding RNAs (ncRNAs) such as microRNAs (miRNAs) and long non-coding RNAs (lncRNAs) [15,16]. Although the development of ectodermal tissues and associated diseases is largely governed by genetic and molecular pathways, growing evidence suggests that epigenetic mechanisms also play critical roles in the pathogenesis of ectodermal disorders [20,21,22,23,24]. Similarly, epigenetic alterations have been implicated in the ageing process, further reinforcing their biological importance [25]. It is therefore reasonable to investigate the potential contribution of epigenetic mechanisms in syndromes such as NF1 and NF2. Epigenetic dysregulation has been linked to various neurological and oncological disorders, including gliomas, medulloblastomas, and malignant peripheral nerve sheath tumours [26,27,28,29]. Early studies in NF1- and NF2-associated tumours have revealed abnormal patterns of DNA methylation, dysregulation of expression, and disruptions in chromatin regulation, suggesting that these changes may influence tumour suppressor gene function, Schwann cell behaviour, and the tumour microenvironment, ultimately contributing to the clinical heterogeneity observed in these disorders [30,31].

While numerous studies have explored the molecular basis of NF1 and NF2, research on the role of epigenetic mechanisms remains fragmented. The absence of an integrated model has created the need for a comprehensive review, aiming to organise the existing knowledge and highlight its clinical relevance. The current review seeks to critically evaluate the role of epigenetic mechanisms in the pathogenesis and prognosis of NF1 and NF2, presenting emerging evidence that supports these relationships. Specifically, we discuss the impact of DNA methylation, histone modifications, and ncRNA on disease expression and tumour behaviour, emphasising their contribution to the complexity of neurofibromatosis. Understanding epigenetic regulation in these syndromes is crucial, as epigenetic markers hold potential for prognostic evaluation, guiding therapeutic decisions, and developing targeted therapies.

## 2. The Epigenetic Landscape of NF1

Epigenetic mechanisms are increasingly recognised as important contributors to the biological complexity of NF1. Although NF1 is primarily caused by germline mutations in the *NF1* tumour suppressor gene, growing evidence suggests that epigenetic alterations significantly contribute to disease expression. Epigenetic modifications, including DNA methylation, histone alterations, miRNAs, and lncRNA, have been implicated in the pathogenesis of NF1, as well as in the regulation of tumour initiation, progression, and phenotypic heterogeneity, among NF1-associated neoplasms. These alterations may influence disease expression independently or in conjunction with genetic mutations. In some cases, they may mimic or enhance the effects of a second genetic hit, contributing to NF1 manifestation. As such, epigenetic mechanisms may help explain the variability in NF1 pathogenesis and prognosis beyond its underlying genetic mutations.

### 2.1. An Overview of NF1

NF1 is a complex genetic disorder inherited in an autosomal dominant manner caused by germline mutations in the *NF1* tumour suppressor gene located on chromosome 17q11.2 [32]. The gene product, neurofibromin, functions primarily as a negative regulator of the Ras signalling pathway but also contributes to tumour suppression through additional mechanisms [33]. NF1 affects approximately 1 in 2500 to 3500 individuals worldwide, with no clear differences across ethnic or racial groups [12]. Clinically, NF1 presents with a broad spectrum of manifestations, including characteristic pigmentary changes such as café-au-lait macules and intertriginous freckling [34,35]. Neurofibromas, a hallmark of the disease, are classified into distinct subtypes: cutaneous neurofibromas (CNFs), subcutaneous neurofibromas, and plexiform neurofibromas (PNFs). Additional common features include Lisch nodules of the iris, learning disabilities, skeletal abnormalities, and optic pathway gliomas [36,37,38,39]. Malignant peripheral nerve sheath tumours (MPNSTs), formerly known as neurofibrosarcomas, represent one of the most severe complications associated with NF1 [33,40,41]. The lifetime risk of developing an MPNST in NF1 is 8–13%. As the most lethal malignancy arising in individuals with NF1, MPNSTs are a leading cause of disease-related morbidity and mortality.

These high-grade sarcomas most commonly develop through the malignant transformation of pre-existing PNFs, though they may also arise from deep-seated spinal or subcutaneous lesions. NF1-associated MPNSTs are typically more aggressive than their sporadic counterparts, exhibiting an earlier onset, increased local invasiveness, and a higher propensity for metastasis, ultimately contributing to a markedly poor prognosis in patients with NF1. In addition, a small subset of NF1 patients develop high-grade astrocytomas with piloid features (HGAPs), a rare and aggressive type of brain tumour [30]. While a single mutation in the *NF1* gene initiates the disorder, the variability in the clinical manifestations among patients is considerable [42]. This phenotypic diversity is believed to result from complex interactions involving multiple cell types, cellular signalling pathways, and the extracellular matrix. Importantly, the development of certain NF1-related features, particularly tumour formation, often requires a second somatic mutation, or a “second hit”, leading to bi-allelic inactivation of *NF1* in specific cell populations. Epigenetic mechanisms such as DNA methylation and histone modifications may contribute to or mimic this second-hit event by functionally silencing the remaining wild-type *NF1* allele, thereby facilitating tumour initiation and progression.

### 2.2. The Role of Epigenetic Mechanisms in NF1 Pathogenesis

#### 2.2.1. DNA Methylation

Epigenetic alterations, particularly DNA methylation, are increasingly implicated in the pathogenesis of NF1. In this regard, bisulphite modification of DNA was performed on leukocyte samples from 79 NF1 patients and 79 healthy controls to evaluate the methylation status of the mismatch repair genes *MLH1*, *MSH2*, *MSH6*, and *PMS2* using methylation-specific PCR and pyrosequencing [43]. No methylation in the *MLH1*, *MSH6*, and *PMS2* promoters in the blood DNA of either the controls or the NF1 patients was found. However, *MSH2* promoter methylation near transcription start points was detected in both the control samples and the NF1 samples, but significantly higher levels of methylation were observed in the NF1 group. Within the NF1 population, increased methylation at two out of six CpG sites in the *MSH2* promoter was particularly evident in patients exhibiting a high burden of cutaneous neurofibromas. Therefore, differences in the methylation of the *MSH2* gene may modulate mismatch repair capacity and act as an epigenetic modifier contributing to phenotypic variability in NF1. The reduced DNA repair efficiency resulting from *MSH2* methylation may promote the accumulation of somatic mutations, thereby contributing to NF1 pathogenesis.

Further elaborating on this point, methylation profiling of 45 CNFs, 17 PNFs, and 9 normal skin and nerve samples from NF1 patients using the Illumina EPIC 850K array revealed significant chromatin conformational differences between the CNFs and PNFs [44]. These differences were strongly associated with region-specific methylation events, including two differentially methylated regions located upstream of the *MAP2K3* transcription start site. This variation in methylation corresponded to increased protein expression of MKK3 and p38 in the CNFs and decreased expression in the PNFs. Since the MKK3/p38 axis is involved in RAS-mediated activation of stress and inflammatory responses, CNFs appear to be epigenetically primed toward a pro-inflammatory and chromatin remodelling state. In contrast, PNFs favour RAS/MEK/ERK pathway activation, supporting proliferative signalling. The CpG methylation analysis also identified 34 sites significantly associated with CNF tumour size. Although the corresponding genes of these sites could not be confidently mapped with statistical confidence and no pathway associations were confirmed, the findings suggest that these methylation events influence growth through currently unrecognised mechanisms. The distinct methylation patterns observed between CNFs and PNFs contribute to divergent signalling profiles, providing a mechanistic explanation for their differing biological behaviour and pathogenesis without invoking differences in genetic mutations or histology.

**Figure 1:**
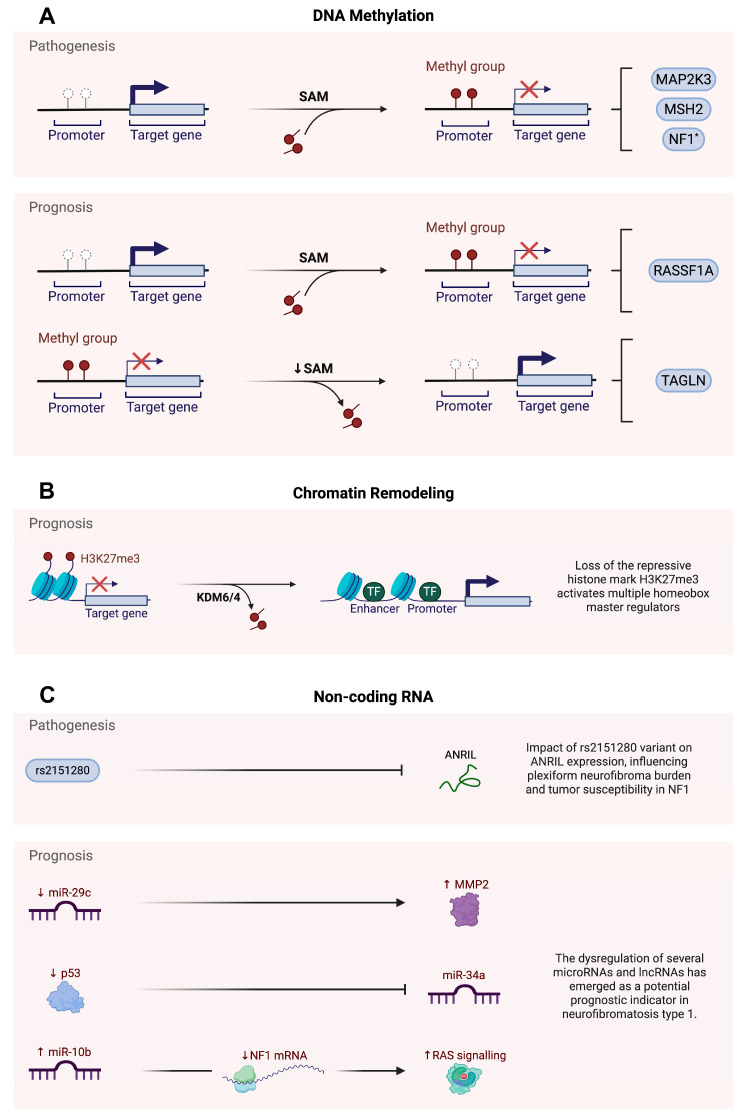
Epigenetic regulation of pathogenesis and prognosis in neurofibromatosis type 1. (**A**) DNA methylation, (**B**) chromatin remodelling, (**C**) non-coding RNA. * Although NF1 promoter methylation has been implicated in pathogenesis of NF1, several studies have reported that such methylation events may not play a significant role in disease development. SAM: S-adenosylmethionine; MAP2K3: mitogen-activated protein kinase 3; MSH2: MutS homolog 2; NF1: neurofibromatosis type 1; RASSF1A: Ras association domain family member 1 isoform A; TALGN: transgelin; H3K27me3: trimethylation of lysine 27 on histone H3; KDM6: lysine demethylase 6; KDM4: lysine demethylase 4; TF: transcription factor; rs2151280: reference SNP ID 2151280; ANRIL: antisense non-coding RNA in the INK4 locus; MMP2: matrix metalloproteinase 2; p53: tumour suppressor p53; RAS: rat sarcoma virus oncogene; miR-29c: microRNA-29c; miR-34a: microRNA-34a; miR-10b: microRNA-10b; mRNA: messenger ribonucleic acid.

Methylation may also contribute to phenotypic diversity in NF1, as shown by the analysis of eight monozygotic pairs of twins with the disorder [45]. To investigate whether epigenetic changes in the *NF1* promoter could explain differences in clinical features—such as optic glioma—DNA from peripheral blood leukocytes was analysed. Methylation patterns were mapped across 900 base pairs of the *NF1* promoter region. All pairs of twins showed clear differences in methylation, especially in regulatory regions like the 5′ untranslated region (UTR), exon 1, intron 1, transcription factor binding sites, and the NF1HCS promoter element. In the −249 to −234 base pair region of the promoter, pairs of twins who differed in optic glioma status showed greater methylation differences than those in twins who were both affected or unaffected. In these discordant pairs, the twin without glioma had lower methylation at this region than that in their affected sibling. These findings suggest that even with identical DNA, changes in methylation within regulatory areas of the *NF1* gene may alter gene expression, leading to different clinical outcomes, such as optic glioma (Figure 1A). This highlights the role of methylation as a potential modifier of NF1 pathogenesis and phenotype.

While several studies emphasise the role of methylation in modifying NF1 tumour behaviour and phenotype, other findings indicate that promoter methylation of the *NF1* gene itself is not a primary driver of gene silencing [46,47,48,49]. The methylation patterns of the *NF1* promoter region were evaluated through bisulphite genomic sequencing [46]. Tissue samples from NF1 patients, including dermal neurofibromas, PNFs, and MPNSTs, were collected, along with leukocyte samples from healthy individuals without NF1. There was no evidence of global hypermethylation in the *NF1* promoter region across NF1-associated tumours. Additional evidence from an analysis of 20 neurofibromas and 3 neurofibrosarcomas derived from 23 patients, of whom 17 had been diagnosed with NF1, supports the above [47]. Loss of heterozygosity of the *NF1* gene region and microsatellite instability were investigated using specific markers. The methylation patterns of the SP1 binding site and the CRE site near the transcription start site of the *NF1* gene were studied using methylation-specific PCR. There was no evidence of microsatellite instability or *NF1* promoter methylation in any of the tumours.

Consistent findings emerged from examining NF1-related PNFs lacking detectable somatic *NF1* mutations and healthy Schwann cell samples [48]. While more than half of the tumours exhibited some degree of methylation, this pattern was attributed to the heterozygous background characteristic of NF1 tumours. The majority of the tumour samples did not display significantly higher methylation than that in the healthy Schwann cell controls. Moreover, the analysis did not reveal widespread methylation across the *NF1* promoter region in any of the samples. These results indicate that although localised methylation levels are present, they do not reach a threshold that would support a primary role in *NF1* gene silencing. Lastly, a study utilising bisulphite-modified genomic sequencing examined the methylation pattern of the *NF1* promoter region in tissue samples obtained from NF1-specific tumours and peripheral blood lymphocytes from NF1 patients and healthy controls [49]. CpG methylation specific to the tumour was detected at six different positions; these were −609, −429, −406, −383, −331, and −315 in relation to the transcription start site. However, the remaining CpG sites investigated were unmethylated across all of the other tissue samples studied, including both the NF1-related tumour samples and the peripheral blood leukocytes from the NF1 patients and healthy controls. This suggests that CpG hypermethylation within the *NF1* promotor is not likely to be the contributing genetic factor leading to the development of neurofibromas.

#### 2.2.2. Non-Coding RNAs

PNFs constitute a major clinical manifestation and phenotypic feature of NF1 [32]. Emerging evidence underscores the pivotal role of epigenetic regulators, including lncRNA such as ANRIL (antisense non-coding RNA in the INK4 locus), in the pathogenesis of and phenotypic variability in tumour development [50]. Evidence supporting the modifying effect of ANRIL on PNF formation was obtained through high-resolution array comparative genomic hybridisation. The analysis was performed on tissue samples from 22 PNFs collected from 18 NF1 patients with the aim of identifying genetic modifiers involved in tumour development. A family-based association study targeting five cancer susceptibility single-nucleotide polymorphisms (SNPs)—rs1063192, rs2151280, rs2218220, rs10757257, and rs7023329—located within the 9p21.3 chromosomal region was conducted in a cohort comprising 1105 individuals, including 740 patients with NF1 and 365 unaffected relatives. Among these, SNP rs2151280, located within the *ANRIL* gene, was significantly associated with the number of PNFs. A reverse transcription quantitative PCR analysis of the peripheral blood from 124 NF1 patients further demonstrated that the T allele of rs2151280 was linked to reduced ANRIL transcript levels. Notably, the only recurrent somatic alteration identified in the PNF samples was a deletion encompassing the 9p21.3 region, which included the *CDKN2A/B-ANRIL* locus. These findings suggest that ANRIL functions as an epigenetic modifier in NF1, where reduced expression may contribute to increased PNF susceptibility and tumour burden, potentially through modulation of the *CDKN2A/B* tumour suppressor axis.

In a related investigation, the potential association between PNF burden and the SNP rs2151280 within the non-coding RNA gene *ANRIL* was assessed in 29 patients with constitutional NF1 microdeletions [51]. PCR-based genotyping was performed in parallel with clinical and imaging-based assessments of plexiform neurofibroma (PNF) number and volume. Despite previous associations between rs2151280 and tumour burden in the broader NF1 population, this study found no statistically significant relationship between the T allele of rs2151280 and the number or total volume of PNFs in microdeletion patients. These findings suggest that in cases of constitutional *NF1* microdeletions, additional genes such as *SUZ12* are also deleted. As a result, the influence of *ANRIL*, mediated through the rs2151280 variant, may be diminished and is unlikely to represent a significant determinant of disease development (Figure 1C).

### 2.3. The Prognostic Impact of Epigenetic Alterations in NF1

#### 2.3.1. DNA Methylation

Hypermethylation has been increasingly associated with prognosis in NF1, particularly in the context of MPNSTs and HGAP [30,31,52]. In these cases, specific methylation patterns, especially hypermethylation of the *NF1* enhancer region, have been identified not only as markers of malignant transformation but also as correlates of poorer clinical outcomes. In relation to this, DNA methylation profiling was performed on 29 tumour samples and adjacent neurofibroma tissue from 13 patients with MPNSTs as well as NF1-MPNST cell lines in order to identify CpG sites specific to malignant transformation [52]. A substantial number of MPNST-specific CpG sites were identified, the majority of which were located in distinct genomic regions that enabled clear differentiation between MPNSTs and adjacent benign neurofibromas. Methylation profiling of plasma samples demonstrated that most of these CpG regions exhibited increased methylation in patients with MPNSTs compared to that in individuals with NF1 who had not developed malignancy. Furthermore, a defined subset of CpG islands successfully distinguished circulating DNA from MPNST patients from that of non-malignant NF1 cases, indicating that tumour-associated hypermethylation patterns are also reflected in the bloodstream. These results suggest that distinct hypermethylation signatures are acquired during malignant transformation in NF1-associated tumours.

Furthermore, *RASSF1A* promoter methylation was observed exclusively in MPNSTs, with no methylation detected in benign neurofibromas or non-neoplastic controls [31]. A total of 113 specimens were analysed, including 44 NF1-associated MPNSTs, 47 sporadic MPNSTs, 21 benign neurofibromas, and 1 non-neoplastic nerve sheath control. Methylation-specific PCR (MSP) and quantitative MSP were used to assess the methylation status of the *RASSF1A* promoter. Methylation of *RASSF1A* occurred exclusively in the malignant samples, with approximately 60% of the MPNSTs showing promoter methylation, while all benign and control samples remained unmethylated. Additionally, RASSF1A-methylated tumours demonstrated a significant reduction in *RASSF1A* gene expression compared to that in the unmethylated tumours. Epigenetic silencing of *RASSF1A* may contribute to genomic instability, including increased DNA copy number alterations and complex chromosomal rearrangements, hallmark features of MPNSTs. The survival analysis indicated that in NF1-associated MPNSTs, *RASSF1A* promoter methylation serves as a marker of a poor prognosis, independent of other clinical risk factors such as tumour size and metastasis. These findings suggest that RASSF1A promoter hypermethylation is an important epigenetic factor in the clinical progression of NF1-associated MPNSTs (Figure 1A). In addition to these methylation signatures, recent evidence suggests that telomere attrition may also contribute to MPNST pathogenesis, potentially through epigenetic mechanisms such as altered methylation of subtelomeric regions and histone modifications at telomeric chromatin [53,54,55]. However, this possibility remains to be thoroughly investigated. A longitudinal whole-exome study of NF1-associated tumour evolution revealed progressive NF1 inactivation accompanied by telomere-associated genomic instability, highlighting telomere shortening and dysregulation of the shelterin complex as possible contributors to malignant transformation [53].

Methylation-driven mechanisms have also been shown to influence the prognosis in NF1-associated HGAP [30]. A tumour analysis in 148 patients identified three distinct HGAP subgroups through whole-genome DNA methylation profiling. One subgroup, termed gNF1, was characterised by a unique epigenetic signature involving hypermethylation of the *NF1* enhancer region. Clinically, the tumours within the gNF1 subgroup were associated with an unfavourable prognosis, characterised by a predilection for the posterior fossa, a more aggressive progression rate, and significantly reduced overall survival. The gNF1 tumours also exhibited a distinctive microenvironment enriched with non-neoplastic glial and neuronal cells, further correlating with their aggressive behaviour. Moreover, the associated epigenetic changes were linked to altered RNA processing, which may contribute to unfavourable clinical outcomes. These findings suggest that hypermethylation of the *NF1* enhancer region is associated with a poorer prognosis in NF1 patients, as it defines a subgroup of tumours with more aggressive behaviour, faster progression, and reduced overall survival.

While the hypermethylation of tumour suppressor genes has been associated with a malignant transformation and poor prognosis in NF1-associated MPNSTs, evidence also highlights the role of gene-specific hypomethylation in promoting oncogenic signalling pathways [56]. Tumour samples were obtained from PNFs and MPNSTs from eight NF1 patients to investigate the epigenetic regulation of the *TAGLN* gene, which encodes transgelin, an actin-associated protein. DNA was extracted, bisulphite-treated, and analysed using methylation-specific PCR and array-based profiling to assess the methylation in the promoter and sub-promoter regions of *TAGLN*. The transgelin expression was significantly upregulated in NF1-associated MPNSTs compared to plexiform neurofibromas, and this overexpression corresponded with hypomethylation in *TAGLN* regulatory regions. To examine the functional relevance of this finding, *TAGLN* expression was experimentally manipulated. TAGLN knockdown in MPNST cells resulted in decreased RAS and ERK1/2 signalling, while TAGLN overexpression in non-malignant NF1 cells enhanced the activation of these pathways. These results demonstrate that hypomethylation-driven upregulation of *TAGLN* promotes RAS-MAPK signalling, contributing to the malignant transformation and progression of NF1-associated peripheral nerve sheath tumours.

#### 2.3.2. Histone Modifications

Beyond DNA methylation, dysregulation of histone modification, particularly the loss of histone H3 lysine 27 trimethylation (H3K27me3), has emerged as a key epigenetic mechanism driving the development of MPNSTs and shaping the clinical prognosis in individuals with NF1, largely through disruption of the Polycomb repressive complex 2 (PRC2) and its failure to repress critical developmental and proliferative genes [57,58,59]. An analysis of 15 MPNSTs from 12 patients, including 6 NF1-associated tumours, 4 sporadic tumours, 4 radiotherapy-associated tumours, and 1 epithelioid MPNST, was conducted [57]. Using whole-exome sequencing, copy number profiling, and RNA sequencing revealed recurrent loss-of-function mutations in core PRC2 components, namely *EED* and *SUZ12*. These mutations were detected in 92% of sporadic tumours, 70% of NF1-associated tumours, and 90% of radiotherapy-associated tumours. These genetic inactivations were consistently associated with loss of the H3K27me3 epigenetic mark. The absence of this repressive modification led to the aberrant expression of developmental regulators, including homeobox (HOX) transcription factors. The reintroduction of functional PRC2 components restored H3K27me3 levels and reduced cell proliferation, indicating its role in tumour suppression. These findings highlight PRC2 inactivation and H3K27me3 loss as central events in MPNST tumourigenesis, with important implications for the epigenetic prognosis in NF1 (Figure 1B).

Additional evidence supporting the role of histone modification in MPNSTs was provided through an H3K27me3 immunohistochemical analysis of tissue microarrays comprising 162 primary MPNSTs, 97 neurofibromas, and 341 other soft tissue tumours [58]. Loss of H3K27me3 was observed in 34% of the MPNSTs, while all neurofibromas, including atypical and plexiform, retained H3K27me3 expression. Importantly, H3K27me3-negative MPNSTs were associated with a significantly worse overall survival, a finding confirmed across two independent patient cohorts. While H3K27me3 immunohistochemistry shows utility as a diagnostic marker, its limited specificity precludes reliable differentiation of MPNSTs from other histologically similar sarcomas. Further validation of H3K27me3 as a prognostic marker in MPNSTs was achieved through an immunohistochemical analysis of 100 MPNSTs, including 70 sporadic, 10 NF1-associated, 10 radiation-associated, and 10 epithelioid cases [59]. These were compared against 200 histological mimics, including spindle cell sarcomas and benign schwannoma tumours. Loss of H3K27me3 expression was observed in 51% of all MPNSTs; however, none of the epithelioid MPNSTs exhibited this loss. Among the MPNSTs, H3K27me3 loss correlated strongly with tumour grade, being present in 29% of low-grade, 59% of intermediate-grade, and 83% of high-grade tumours. These findings highlight PRC2 inactivation and H3K27me3 loss as central epigenetic events in MPNST pathogenesis, with strong and prognostic relevance in NF1-associated tumours. Beyond their prognostic implications, histone modifications may also play a role in NF1 pathogenesis and therapeutic targeting, potentially influencing treatment responses and disease progression [60,61]; the literature on this topic remains scattered and comparatively limited.

#### 2.3.3. Non-Coding RNAs

Disruption of miRNA pathways has also been implicated in the pathogenesis and poor prognosis of NF1-associated MPNSTs [62,63,64]. A total of 97 frozen tumour samples were analysed, including 20 MPNSTs, 37 neurofibromas, 27 schwannomas, and 13 synovial sarcomas [62]. Global mRNA and microRNA expression profiling using microarray platforms revealed that MPNSTs exhibit widespread downregulation of gene expression, with prominent loss of the tumour suppressor miR-34a. Functional studies in MPNST cell lines have demonstrated that p53, a key upstream regulator of miR-34a, is frequently inactivated in these tumours, thereby directly contributing to the reduced expression of miR-34a. Restoration of either p53 or miR-34a in the MPNST cells induced apoptotic cell death, demonstrating their tumour-suppressive function. These findings highlight that p53-mediated repression of miR-34a represents a significant epigenetic mechanism driving malignant progression in MPNSTs and may play a role in the poor clinical outcomes observed in NF1 patients (Figure 1C).

Expanding the understanding of the involvement of microRNA in MPNST pathogenesis, miR-10b has also been identified as a key regulator in NF1-associated tumour progression and prognosis [63]. NF1-associated MPNST cell lines, primary Schwann cells derived from dermal neurofibromas and PNFs, and healthy human Schwann cells were analysed using microarray profiling and quantitative real-time PCR. In addition, the Ewing sarcoma cell line (SK-ES1) was included in the analysis. The miR-10b expression was significantly elevated in the MPNST cells and tumour tissues compared to that in the benign neurofibromas and normal controls. Functional assays demonstrated that miR-10b directly targets the 3′ UTR of *NF1* mRNA, leading to the suppression of neurofibromin, a key negative regulator of RAS signalling. In SK-ES1 cells, miR-10b inhibition restored neurofibromin expression and reduced RAS activity. Similarly, antisense inhibition of miR-10b in the NF1 MPNST cell lines led to decreased cell proliferation, migration, and invasion. These findings indicate that miR-10b contributes to tumourigenesis in NF1 by promoting oncogenic RAS signalling through epigenetic repression of neurofibromin, thereby enhancing malignant behaviour and potentially impacting clinical outcomes.

Further support for the role of microRNA dysregulation in MPNST aggressiveness comes from the analysis of miR-29c [64]. Tissue samples from neurofibromas and NF1-associated MPNSTs were analysed using microarray profiling, followed by validation with reverse transcription quantitative PCR. The differential expression analysis identified 16 microRNAs with altered levels between tumour types—14 of which were downregulated in MPNSTs and 2 of which were upregulated compared to neurofibromas. Among the downregulated candidates, miR-29c was selected for further investigation due to its consistent and significant reduction in the malignant samples. Functional assays using MPNST cell lines transfected with miR-29c mimics revealed that its overexpression significantly reduced cell invasion without affecting proliferation. The target analysis identified several extracellular-matrix-related genes, including *matrix metalloproteinase 2* (*MMP2*), as downstream effectors of miR-29c. The suppression of MMP2 expression following miR-29c upregulation confirmed its role in modulating invasive behaviour. These findings demonstrate that miR-29c acts as a tumour-suppressive microRNA in NF1, where its epigenetic downregulation enhances MPNST invasiveness via MMP2-mediated pathways. This increased invasive capacity may contribute to a more aggressive tumour phenotype and a poorer clinical prognosis in affected individuals.

## 3. The Epigenetic Architecture of NF2

Alterations such as promoter methylation and miRNA activity have been implicated in modulating gene expression and signalling pathways in NF2. In certain contexts, these epigenetic modifications may cooperate with genetic mutations or act independently to promote tumourigenesis, affecting cellular processes such as proliferation, apoptosis, and invasion. Although the prevalence and consistency of these alterations remain under investigation, epigenetic regulation may offer additional insight into the heterogeneity of NF2-related tumour behaviour and represent a potential target for future therapeutic strategies.

### 3.1. An Overview of NF2

NF2-related schwannomatosis (NF2-SWN), formerly known as neurofibromatosis type 2, is an autosomal dominant disorder associated with the presence of bilateral vestibular schwannomas (VSs), meningiomas, and distinctive nervous system lesions [65,66,67]. NF2 results from loss-of-function alterations in the *NF2* gene on chromosome 22, with resultant dysfunction in its protein product merlin. Bilateral VSs are the traditional pathognomonic diagnostic feature of NF2. However, patients also have a predisposition towards the development of other tumours, including meningiomas and ependymomas, as well as peripheral, spinal, and cranial nerve schwannomas [68,69]. Patients may also develop other characteristic manifestations, such as ocular lesions, neuropathies, meningioangiomatosis, and glial hamartia [70,71,72]. The estimated prevalence of NF2 is 1:50,000, with a birth incidence of 1:28,000. The average age of onset is 18 to 24 years. Almost all affected individuals develop bilateral VSs by the age of 30 years.

Although these tumours are not malignant, their anatomic location and multiplicity lead to great morbidity and early mortality. NF2-associated VSs tend to be more invasive and to have a higher degree of dividing cells than non-NF2 tumours. The average age of death is 36 years [73]. The diagnostic criteria for NF2 have been regularly revised. In 2022, the criteria were reviewed and updated by an international committee of NF experts due to an increasing understanding of clinical and molecular data. NF2-associated tumour formation follows Knudson’s two-hit hypothesis, requiring bi-allelic loss of *NF2*, typically through a germline mutation and a second somatic event. However, *NF2* loss alone may not be sufficient for tumourigenesis, and additional molecular mechanisms may be necessary. In this context, epigenetic alterations, particularly DNA methylation and miRNA dysregulation, have been investigated as potential modifiers of NF2 pathogenesis. However, epigenetic mechanisms may not be the primary drivers of tumour formation in NF2, warranting further investigation [74,75].

### 3.2. Epigenetic Drivers of NF2 Pathogenesis

#### 3.2.1. DNA Methylation

An initial investigation into the epigenetic landscape of NF2-associated schwannomas focused on promoter methylation, revealing that *NF2* gene silencing through promoter hypermethylation may occur in a limited subset of tumours, indicating a rare yet potentially significant mechanism (Table 1). In a comprehensive analysis, the methylation profiles were assessed in a cohort of 44 schwannoma tumour samples, comprising 37 schwannoma tumour samples, 29 from individuals without NF2 and 8 from patients diagnosed with NF2 [76]. In addition to the tumour samples, seven control samples were collected, including blood and normal peripheral nerve sheath and brain tissue. In this study, t-166 and T-153 were anonymised NF2-associated schwannoma samples labelled with alphanumeric codes. These two samples were the only ones among the eight NF2-related tumours that exhibited aberrant *NF2* promoter hypermethylation, indicating that this epigenetic change is rare in this patient subgroup. These findings suggest that *NF2* promoter methylation may contribute to *NF2* gene silencing. However, since only two of the eight NF2-associated tumours showed this aberrant methylation, its role in tumourigenesis appears to be limited to specific cases. Therefore, it was indicated that while epigenetic changes can influence tumour biology in NF2, additional mechanisms are likely involved in most cases, and this warrants further investigation.

In order to investigate the involvement of another potentially significant mechanism that is involved in the epigenetic regulation of the *NF2* gene, a study was conducted to evaluate the *NF2* mRNA expression in NF2-VSs [77]. The *NF2* mRNA expression was analysed in a series of VSs using Northern blotting, including samples from 13 tumours related to NF2. These were compared to RNA from human glioblastoma U-251 MG cells and rat peripheral nerves. Moreover, luciferase reporter assays were employed to test the promoter activity under mutations and artificial methylation. De novo methylation was found in over half of the schwannomas. This methylation status reduced the *NF2* promoter activity by disrupting nuclear protein binding at three CpG sites. Site-specific methylation of these three CpG sites was found within the NF-CAR promoter region, a cis-acting regulatory element in the NF2 gene promoter, which plays a key role in regulating *NF2* gene expression. This methylation substantially suppresses *NF2* expression by disrupting the B1 complex, a previously uncharacterized nuclear protein binding site critical for transactivation. Consistent with this finding, no promoter mutations were found, underlining methylation as a primary gene silencing mechanism. Lastly, it was shown that in vitro proximal CpG island methylation might contribute to *NF2* silencing via chromatin remodelling. The role of aberrant methylation is critical to the pathogenesis of NF2, as it leads to the inactivation of the *NF2* gene, thereby contributing to the development of schwannomas and other tumours in NF2 patients.

Extending beyond promoter silencing, genome-wide methylation profiling has revealed that NF2-associated VSs also exhibit extensive DNA hypomethylation, particularly in oncogenes and regulatory miRNAs, suggesting broader epigenetic dysregulation that may promote tumour development [79]. Infinium Human Methylation 450K BeadChip microarrays were utilized to study the DNA methylation patterns in 36 VS, 4 non-VS, and 5 healthy nerve samples, including NF2-related cases. Genes and microRNAs were verified using a quantitative reverse transcription polymerase chain reaction. The hypomethylation and hypermethylation levels were similar, with a trend toward hypomethylation. Specifically, hypomethylation was exhibited notably at *HOX* genes in several CpG sites in VSs but not in non-VSs. Moreover, in the VS, promoter hypomethylation of miRNA-21, *MET* and *PMEPA1* was observed and found to correlate with increased expression levels based on expression analysis. In NF2 patients, this hypomethylation suggests an epigenetic pathway that enhances oncogenic miRNA and gene expression and potentially could contribute to pathogenesis of the schwannomas.

Diverging from the previously reported evidence, it has been suggested that epigenetic mechanisms, specifically DNA methylation, do not play a significant role in the pathogenesis of NF2-related VSs [68]. An investigation into potential epigenetic and genetic alterations through the collection of tumour tissues and blood samples was conducted in 30 Korean patients with sporadic VSs. Although it was found that 16 cases had *NF2* gene mutations, no aberrant hypermethylation of the *NF2* gene promoter region in patients with VSs was found, and no clear genotype–phenotype correlation was linked to methylation status. This indicates that hypermethylation did not considerably contribute to *NF2* gene silencing in these patients, suggesting that alternative mechanisms such as mutations or deletions have a more crucial role in tumour development in NF2-related VSs.

#### 3.2.2. Non-Coding RNAs

Shifting focus from DNA methylation to ncRNA regulation, miRNA expression profiling in VS has revealed widespread deregulation, underscoring the critical role of miRNAs as epigenetic modulators in the pathogenesis of NF2-related tumourigenesis. The deregulation of miRNAs and other ncRNA was investigated in 16 VSs, including 1 from a patient with NF2. Ten selected miRNAs were validated using quantitative real-time PCR [78]. The NF2-associated schwannoma exhibited a distinct miRNA expression profile compared to that in the sporadic schwannomas and normal nerve tissues. In total, 174 miRNAs were found to be deregulated; specifically, miR-10b, miR-206, miR-183, and miR-204 were significantly downregulated, whereas miR-431, miR-221, miR-21, and miR-720 were markedly upregulated. Among these, miR-10b and miR-204 demonstrated the most pronounced alterations. Furthermore, a distinct cluster of ncRNA located within the 14q32 chromosomal region appeared to be consistently upregulated in VS, suggesting that the dysregulation of miRNAs in this region may contribute to schwannoma pathogenesis. These observations underscore the importance of miRNAs as critical epigenetic regulators in NF2. Their dysregulation appears to contribute to the development or maintenance of schwannomas.

## 4. Conclusions

This review highlights the emerging role of epigenetic mechanisms in the pathogenesis and prognosis of NF1 and NF2. Despite the well-established genetic basis of these syndromes, significant clinical variability remains unexplained, prompting a shift towards exploring additional regulatory layers. Epigenetic alterations, particularly DNA methylation, histone modifications, and the dysregulation of ncRNAs, have been shown to influence key cellular pathways relevant to tumour development, progression, and clinical heterogeneity in NF1 and NF2. Thus, the current evidence supports the notion that these epigenetic mechanisms not only contribute to disease initiation but also modulate prognosis and phenotypic diversity. However, many important questions remain unanswered. Elucidating the role of epigenetic modifications in classic non-tumour manifestations such as café-au-lait macules, freckling, and Lisch nodules remains a key objective. Beyond this, future research must aim to characterize the specific epigenetic changes in NF1 and NF2 associated with distinct clinical manifestations better; clarify their causal role in disease progression; and investigate how epigenetic alterations may influence therapeutic responsiveness and resistance. Moreover, understanding these mechanisms may assist in the development of epigenetically informed treatment strategies tailored to individual patient profiles. Identifying specific epigenetic signatures in individuals with NF1 and NF2 may also inform refinements in the surveillance protocols and ultimately support more targeted clinical monitoring. Overall, integrating epigenetic insights into the study of neurofibromatosis represents a critical step towards a more comprehensive understanding and more personalized management of these complex disorders.

## Figures and Tables

**Table 1:** The epigenetic mechanisms implicated in the pathogenesis of neurofibromatosis type 2.

Epigenetic Mechanism	Key Mediator	Impact	Effect of Modification	Ref.
DNA Methylation	NF2 promoter methylation	Yes	Leads to NF2 gene silencing, which may contribute to tumourigenesis	[76]
DNA Methylation	Methylation of three CpG sites within NF-CAR, a cis-acting regulatory region in the promoter of the NF2 gene	Yes	Inactivation of NF2, which leads to the development of schwannomas and other tumours in NF2 patients	[77]
DNA Methylation	Hypomethylation at HOX genes in several CpG sites; MiRNA-21, MET, and PMEPA1 showed promoter hypomethylation	Yes	Enhances oncogenic miRNA and gene expression; leads to pathogenesis of the vestibular schwannomas	[78]
DNA Methylation	No aberrant hypermethylation of the NF2 gene promoter region was found	No	Alternative mechanisms play a more critical role in the pathogenesis of vestibular schwannomas	[68]
MicroRNAs	MiR-10b, miR-206, miR-183, and miR-204 showed downregulation; miR-431, miR-221, miR-21, and miR-720 showed upregulation; non-coding RNAs in the 14q32 chromosomal region showed upregulation	Yes	Contributes to the development of schwannomas	[79]

DNA: deoxyribonucleic acid; NF2: neurofibromatosis type 2; CpG: cytosine–phosphate–guanine; NF-CAR: Neurofibromatosis Consensus Area of Regulation; HOX: homeobox; MET: MET proto-oncogene, receptor tyrosine kinase; miRNA: microRNA; miRNA-21: microRNA-21; PMEPA1: prostate transmembrane protein androgen induced 1; miR-10b: microRNA-10b; miR-206: microRNA-206; miR-183: microRNA-183; miR-204: microRNA-204; miR-431: microRNA-431; miR-221: microRNA-221; miR-21: microRNA-21; miR-720: microRNA-720; RNA: ribonucleic acid.

## Data Availability

Not applicable.

## Abbreviations

The following abbreviations are used in this manuscript:

ANRIL	Antisense non-coding RNA in the INK4 locus
CDKN2A	Cyclin-dependent kinase inhibitor 2A
CNF	Cutaneous neurofibroma
CpG	Cytosine–phosphate–guanine
CRE	cAMP response element
DNA	Deoxyribonucleic acid
EED	Embryonic ectoderm development
ERK	Extracellular signal-regulated kinase
H3K27me3	Trimethylation of lysine 27 on histone H3
HGAP	High-grade astrocytomas with piloid features
HOX	Homeobox
KDM4	Lysine demethylase 4
KDM6	Lysine demethylase 6
LncRNA	Long non-coding RNA
MAP2K3	Mitogen-activated protein kinase 3
MEK	Mitogen-activated protein kinase
MET	MET proto-oncogene, receptor tyrosine kinase
miRNA	MicroRNA
MKK3	Mitogen-activated protein kinase 3
MLH1	MutL homolog 1
MMP2	Matrix metalloproteinase 2
MPNST	Malignant peripheral nerve sheath tumour
mRNA	Messenger ribonucleic acid
MSH2	MutS homolog 2
MSH6	MutS homolog 6
MSP	Methylation-specific PCR
ncRNA	Non-coding RNA
NF-CAR	Neurofibromatosis consensus area of regulation
NF1	Neurofibromatosis type 1
NF1HCS	NF1 5′ highly conserved sequence
NF2	Neurofibromatosis type 2
NF2-SWN	NF2-related schwannomatosis
p53	Tumour suppressor p53
PCR	Polymerase chain reaction
PMEPA1	Prostate transmembrane protein, androgen induced 1
PMS2	Postmeiotic segregation increased 2
PNF	Plexiform neurofibroma
PRC2	Polycomb repressive complex 2
RAS	Rat sarcoma virus oncogene
RASSF1A	Ras association domain family member 1 isoform A
RNA	Ribonucleic acid
rs2151280	Reference SNP ID 2151280
SAM	S-adenosylmethionine
SK-ES-1	Human Ewing sarcoma cell line
SNP	Single-nucleotide polymorphism
SP1	Specificity protein 1
SUZ12	Suppressor of zeste 12
TALGN	Transgelin
TF	Transcription factor
U-251 MG cells	Uppsala 251 Malignant Glioma cells
UTR	Untranslated region
VS	Vestibular schwannoma
